# Prevalence, morphological and molecular characterization of *Leucocytozoon macleani* (Apicomplexa: Haemosporida) from chickens in Thailand

**DOI:** 10.1051/parasite/2025043

**Published:** 2025-08-05

**Authors:** Nikom Srikacha, Carolina Romeiro Fernandes Chagas, Surya Paudel, Pornchai Pornpanom

**Affiliations:** 1 Department of Animal Science, Faculty of Natural Resources, Rajamangala University of Technology Isan Sakon Nakhon 47160 Thailand; 2 State Scientific Research Institute Nature Research Centre Vilnius 08412 Lithuania; 3 Department of Infectious Diseases and Public Health, Jockey Club College of Veterinary Medicine and Life Sciences, City University of Hong Kong Hong Kong Special Administrative Region PR China; 4 Akkhraratchakumari Veterinary College, Walailak University Nakhon Si Thammarat 80160 Thailand; 5 Informatics Innovation Center of Excellence, Walailak University Nakhon Si Thammarat 80160 Thailand; 6 One Health Research Center, Walailak University Nakhon Si Thammarat 80160 Thailand

**Keywords:** Blood parasite, Co-infection, Domestic chicken, *Leucocytozoon*, Morphology, Prevalence

## Abstract

*Leucocytozoon* species are common in countries with warm climates but are an often neglected blood parasite in poultry. Although *Leucocytozoon macleani* is less virulent than *Leucocytozoon caulleryi*, it can still negatively impact production performance. In Thailand, the available reports indicate a high prevalence of *Leucocytozoon* spp.*,* but detailed morphological characteristics of the parasites remain insufficiently known. In this study, Giemsa-stained blood smears and extracted genomic (g) DNA were obtained from 60 domestic chickens (*Gallus gallus domesticus*). Blood smears were examined for the presence of *Leucocytozoon* species and their morphological characteristics were examined. A total of 60 gDNA samples were used for nested-PCR amplification of the cytochrome *b* gene of *Leucocytozoon* species, followed by sequencing and phylogenetic analysis. The microscopic and molecular examinations revealed prevalence of leucocytozoonosis in chickens of 85% and 90%, respectively. Sequence analysis indicated that several infected chickens harboured multiple *Leucocytozoon* lineages. *Leucocytozoon macleani* was morphologically identified in nine samples and could be linked to the lineages GALLUS17, GALLUS34, and the new lineages GALLUS63. The found gametocytes of *L. macleani* morphologically resembled those reported previously, but exhibited some distinct characteristics. Phylogenetically, the lineages of *L. macleani* isolated in this study grouped separately from some other *L. macleani* lineages deposited in GenBank. In conclusion, the prevalence of *Leucocytozoon* infection in chickens from Northeastern Thailand was high, with frequent co-infections by multiple lineages. *Leucocytozoon macleani* may exhibit cryptic specification. This study is the first report of *L. macleani* lineages described using MalAvi database nomenclature, alongside their morphological characteristics.

## Abstract (fr)

Les espèces de *Leucocytozoon* sont courantes dans les pays à climat chaud, mais sont souvent négligées comme parasites sanguins chez les volailles. Bien que *Leucocytozoon macleani* soit moins virulent que *Leucocytozoon caulleryi*, il peut néanmoins avoir un impact négatif sur les performances de production. En Thaïlande, les rapports disponibles indiquent une prévalence élevée de *Leucocytozoon* spp., mais les caractéristiques morphologiques détaillées des parasites restent insuffisamment connues. Dans cette étude, des frottis sanguins colorés au Giemsa et de l’ADN génomique (g) extrait ont été obtenus chez 60 poulets domestiques (*Gallus gallus domesticus*). Les frottis sanguins ont été examinés pour détecter la présence d’espèces de *Leucocytozoon* et leurs caractéristiques morphologiques ont été étudiées. Soixante échantillons d’ADNg ont été utilisés pour l’amplification par PCR nichée du gène du cytochrome b des espèces de *Leucocytozoon*, suivie d’un séquençage et d’une analyse phylogénétique. Les examens microscopiques et moléculaires ont révélé respectivement une prévalence de leucocytozoonose chez les poulets de 85% et 90%. L’analyse des séquences a indiqué que plusieurs poulets infectés hébergeaient plusieurs lignées de *Leucocytozoon*. *Leucocytozoon macleani* a été identifié morphologiquement dans neuf échantillons et pourrait être lié aux lignées GALLUS17, GALLUS34 et aux nouvelles lignées GALLUS63. Les gamétocytes de *L. macleani* trouvés ressemblaient morphologiquement à ceux rapportés précédemment, mais présentaient certaines caractéristiques distinctes. Phylogénétiquement, les lignées de *L. macleani* isolées dans cette étude se sont regroupées séparément de certaines autres lignées de *L. macleani* déposées dans GenBank. En conclusion, la prévalence de l’infection à *Leucocytozoon* chez les poulets du nord-est de la Thaïlande était élevée, avec de fréquentes co-infections par plusieurs lignées. *Leucocytozoon macleani* pourrait présenter une spécification cryptique. Cette étude est le premier rapport sur les lignées de *L. macleani* décrites à l’aide de la nomenclature de la base de données MalAvi, ainsi que sur leurs caractéristiques morphologiques.

## Introduction

*Leucocytozoon* species (Apicomplexa: Haemosporida) are exclusively bird parasites [64]. They can be distinguished from other haemosporidian parasites, such as *Plasmodium*, *Haemoproteus*, by the absence of pigment granules (haemozoin) [63]. Merogony of *Leucocytozoon* species does not take place in blood cells; only gametocytes are present in blood cells. *Leucocytozoon* species are heteroxenous parasites, meaning their life cycles involve both birds and insect vectors (blackflies for almost all *Leucocytozoon* spp. and biting midges for *Leucocytozoon caulleryi*) [58]. Sexual process and sporogony occur in the insects, while asexual reproduction (merogony or schizogony), and production of gametocytes occur in avian hosts [3]. Infections with *Leucocytozoon* spp. in birds results in a disease called leucocytozoonosis [59].

About 45 species of *Leucocytozoon* have been described [58, 64]. Among these, three species – *L. caulleryi*, *L. macleani* (syn. *L. sabrazesi*) and *L. schoutedeni* (syn. *L. andrewsi, L. gallinarum*) – are found in domestic chickens (*Gallus gallus domesticus*) [48]. *Leucocytozoon caulleryi* is well known for its high virulence, causing fatal haemorrhagic disease in chickens [36]. Haemorrhage may result from the rupture of megalomeronts, which can be developed in many organs, including the pectoral muscle, spleen, liver, intestine, pancreas, kidney, heart, lung and even eyes [11, 50, 56]. In the female reproductive system, large meronts can be found in the ovary and oviduct, leading to inflammation and atrophy in the reproductive tract, and ultimately a drop in egg production and shell-less eggs [30]. Additionally, *L. caulleryi*-infected laying hens may exhibit anaemia and green diarrhoea [31]. In contrast, *L. macleani* and *L. schoutedeni* are less pathogenic, but can still lead to decreased productivity (both egg and meat), anaemia, and green diarrhoea [58].

In Southeast Asia, chicken leucocytozoonosis has been reported in Vietnam, Myanmar, Indonesia, Malaysia, Philippines, Singapore and Thailand [13, 26, 28, 66]. In Thailand, the first case of chicken leucocytozoonosis, known as Bangkok haemorrhagic disease, was reported in 1945 [7, 25]. In recent years, the agents of leucocytozoonosis reported in this region are mainly less virulent species such as *L. sabrazesi* and *Leucocytozoon* sp. [5, 25, 35, 53]. Also, cases of pathogenic *L. caulleryi* have also been found [36], as well as cases of *L. schoutedeni*, lineages GALLUS06 and GALLUS07, which was molecularly detected from backyard chickens in Thailand [5]. However, identification of *Leucocytozoon* species is mainly based on the morphological features of their blood stages (gametocytes) and vertebrate-host specificity data [64]. Thus, confirmation of *L. schoutedeni* infection in Thai chickens requires further investigation, preferably combining microscopic and molecular techniques, which will help to shed light on the transmission of *L. schoutedeni* in Thailand.

A fragment of 479 bp of the cytochrome *b* (*cytb*) gene is widely used as DNA barcoding fragment for *Leucocytozoon* species detection worldwide. Currently, approximately 1,542 *Leucocytozoon* lineages are deposited in the MalAvi database (http://130.235.244.92/Malavi/), an online open access database of *cytb* sequences of haemosporidian parasites [4]. In domestic chickens, there are about 50 described lineages of *Leucocytozoon* species, primarily from Asia and Africa [4]. Among these, several lineages were not identified to the species level. Additionally, a previous phylogenetic study [5] revealed that some *Leucocytozoon* resembling *L. macleani* are grouped separately from *L. macleani*. This information highlights the high genetic diversity within *Leucocytozoon* and underscores the challenges in identifying new parasite lineages or previously overlooked species in chickens.

The active transmission of chicken leucocytozoonosis in Thailand suggests that the disease remains a significant concern for poultry health and production. Even though the less pathogenic parasite species were reported, continuous monitoring is needed to prevent their negative impacts [53]. Additionally, information on tissue merogony and damage of internal organs by *Leucocytozoon* parasites remains fragmentary, but is important for better understanding the mechanisms of pathology during chicken leucocytozoonosis. Takang *et al*. (2017) suggested that research on *Leucocytozoon* spp. in Thai chickens is incomplete, and novel lineages and species are potentially overlooked. This gap may hinder a comprehensive understanding of genetic diversity, host-parasite interactions, pathology, and the development of treatment and prevention strategies. Thus, this study aimed to contribute new knowledge to better understand the distribution and identification of *Leucocytozoon* species and lineages infecting village chickens in Northeastern Thailand, using microscopic and molecular techniques.

## Materials and methods

### Ethical considerations

All sampling procedures in animals were reviewed and approved by the Walailak University Institutional Animal Care and Use committee (Approval number: WU-ACUC-66091). Handling of samples and molecular analysis of blood parasites were conducted complying with the regulation of the Institutional Biosafety Committee (IBC) of Walailak University (Approval number: WU-IBC-66-066).

### Blood collection and sample processing

**Figure 1:**
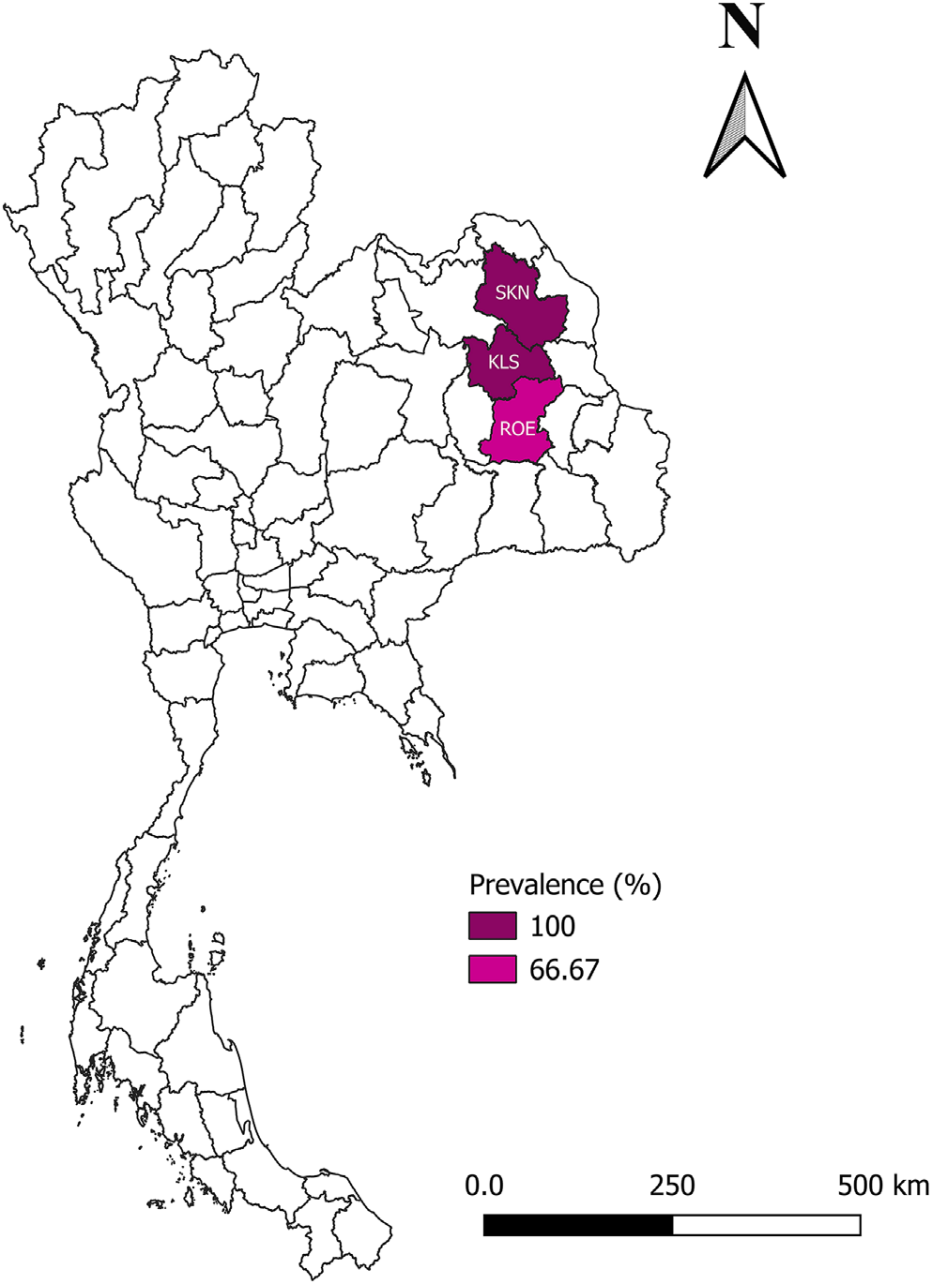
Study sites and prevalence of *Leucocytozoon* species in domestic chickens in Northeastern provinces of Thailand. Kalasin (KLS), Roi-Et (ROE), and Sakon Nakhon (SKN). The figure is generated by QGIS version 3.36.2-Maidenhead.

In March 2024, during the dry season with low rainfall [32], 60 blood samples (0.5 mL) were collected from village chickens (male = 41 and female = 19) raised in Kalasin (*n* = 27), Roi-Et (*n* = 18), and Sakon Nakhon (*n* = 15), Northeastern Thailand (Fig. 1). Fresh blood smears were prepared in triplicate immediately after blood collection. The blood smears were air-dried, fixed in absolute methanol for one minute, and stained with 10% Giemsa solution for one hour [60]. The remaining blood was transferred into an ethylenediaminetetraacetic acid (EDTA) tube (MediPlus^TM^, Bangkok, Thailand) and transported to the Laboratory of Veterinary Clinical Pathology, Akkhraratchakumari Veterinary College, Walailak University, maintaining cold-chain transportation. Afterwards, the samples were kept at 4 °C until further molecular analysis was performed.

### Microscopic examination and parasite morphology

Giemsa-stained blood smears were examined for the presence of *Leucocytozoon* parasites as well as other blood parasites using an Olympus BX43 light microscope (Olympus, Tokyo, Japan) equipped with a digital camera (OlympusDP27, Olympus) and CellSens imaging software (version 1.18, Olympus). The smears were examined at 400× magnification across 100 fields and then re-evaluated at 1000× magnification for 100 fields [60]. Parasitaemia of *Leucocytozoon* was estimated as a percentage by actual counting of the number of parasites per 10,000 red blood cells [61] and categorised as not infected (no detectable parasites), low (<0.1%), moderate (0.1%–1%), and high (>1%) [65]. Additionally, samples that were PCR-positive for *Leucocytozoon* sp. were used for morphometric analyses.

Images of macrogametocytes and microgametocytes, captured from samples with parasitaemia ranging from 0.03–0.16%, were analysed using CellSens imaging software (version 1.18, Olympus), following previous protocols [14, 48, 57, 61]. Measurements were conducted on gametocytes of two lineages of *L. macleani*, GALLUS17 and GALLUS63. For GALLUS17, parasitic forms included both fusiform and roundish macrogametocytes and microgametocytes. In contrast, for GALLUS63, only fusiform macrogametocytes and microgametocytes were included, as roundish forms were rarely observed.

### Genomic DNA extraction and nested-PCR

Genomic (g) DNA was extracted from 50 μL of all 60 blood samples, using a Blood Genomic DNA Extraction Mini Kit, following manufacturer’s recommendation (FavorPrep, Pingtung, Taiwan). The initial PCR utilised primers HaemNFI and HaemNR3 [20] to amplify parasite mitochondrial DNA of haemosporidian parasites (*Haemoproteus* sp., *Plasmodium* sp. and *Leucocytozoon* sp.). In all, 2 μL of the initial PCR product were used for the second PCR, using primers HaemFL and HaemR2L which amplify 479 bp (excluding primers) of *Leucocytozoon* spp.

The PCR mix was prepared in a total volume of 20 μL containing 10 μL of PCR master mix (OnePCR^TM^ Ultra, Bio-Helix, New Taipei City, Taiwan), 1 μL of each primer (concentration = 10 μM), 6 μL of distilled water and 2 μL of DNA template (concentration < 25 ng/μL). *Leucocytozoon* sp. strain KU483 [27] and distilled water were used in every run as positive and negative controls, respectively. Thermal profile of nested-PCR was followed as described in the original protocol [20]. The amplicons were visualised by electrophoresis in 1.5% agarose gel containing the nucleic acid staining (RedSafe^TM^, iNtRON Biotechnology, Korea). Finally, the positive bands were cut and submitted to the U2Bio Thailand (Bangkok, Thailand) for gel extraction, DNA purification and Sanger sequencing.

### Sequence and phylogenetic analysis

Sequences of *Leucocytozoon* sp. were analysed using BioEdit, version 7.0.5.3 [18]. Electropherograms were checked for the presence of double peaks, indicating co-infection [5], which were excluded for subsequent sequence and phylogenetic analysis. Of note, identification of lineages from multiple strains was not available in this study. Only samples showing single infections had their forward and reverse strands aligned to obtain consensus sequences. The consensus sequences (479 bp) were compared with other known lineages deposited in the MalAvi database [4] using BLAST. A sequence with at least one nucleotide difference was identified as a new lineage [5, 9]. The new lineage was then named and deposited in the MalAvi database. If the analysed sequences showed 100% match with known lineages, they were named as their identical lineage. All the sequences from this study were deposited in GenBank (accession numbers PQ560893, PQ560895, PQ560899, PQ560906-08, PQ560910, PQ560911, PQ560916, and PQ880117).

For Bayesian phylogenetic analysis, 32 *cytb* sequences of *Leucocytozoon* sp. deposited in MalAvi database [4] and four sequences from this study were used. The phylogenetic tree was rooted with sequences of *Haemoproteus columbae* and *Haemoproteus iwa*. Bayesian phylogenetics was calculated with MrBayes, version 3.2.6 [44], and the analysis was run for three million generations and every 100 trees were sampled. The first 25% of trees were discarded as burn-in and the remaining trees (37,500) were used to generate the consensus tree. According to the hierarchical likelihood ratio tests (hLRTs), the best substitution model for all alignments was general time-reversible (GTR). Genetic divergence was calculated using the Jukes-Cantor model, implemented in MEGA11 [54].

### Statistical analysis

The prevalence of *Leucocytozoon* sp. was calculated based on the results of both microscopic and molecular analysis. The confidence intervals (95% CI) were calculated using the function “*binom.approx*” in the “*epitools*” package. Logistic regression was applied to determine the associations between prevalence of *Leucocytozoon* sp.-infected chickens with sex (male and female) and localities (Kalasin, Roi-Et and Sakon Nakhon). Significance was obtained at a *p* < 0.05. Student’s *t*-test was used to evaluate the morphometric differences of fusiform gametocytes between the GALLUS17 and GALLUS63 lineages, with statistical significance determined at *p* < 0.05. All statistical analyses were implemented in R, version 4.3.0 [43].

## Results

### Prevalence of *Leucocytozoon* infection

Blood smear examination revealed 85% (95% CI: 75.97%–94.03%) prevalence of *Leucocytozoon* infection in chickens (Table 1). The nested-PCR showed slightly higher prevalence of 90% (95% CI: 82.41%–97.59%). Generally, most of the infected chickens had a moderate level of parasitaemia, ranging from 0.01% to 0.16% in 82.35% of the infected chickens. Eight infected chickens had low parasitaemia (<0.01%) and one infected chicken had high parasitaemia (1.77%). Based on microscopic examination, *Leucocytozoon* sp. (gametocytes in fusiform host cells) and *L. macleani* were present in 40 and 9 individuals, respectively.

**Table 1:** Microscopic examination of parasite infection in chickens in Thailand.

Parasites	Sex	Positive samples	Negative samples	Prevalence (95% CI)
*Leucocytozoon* sp.	Male	36	5	85% (75.97%–94.03%)
	Female	15	4	
	Total	51	9	
*Plasmodium* sp.	Male	1	40	1.67% (0.00%–4.91%)
	Female	0	19	
	Total	1	59	
*Trypanosoma* sp.	Male	5	36	15.00% *(5.97%–24.03%)
	Female	4	15	
	Total	9	51	

*Prevalence of trypanosomes was previously reported by Pornpanom *et al*. [41].

After microscopic examination of 51 fresh blood smear, one fighting cock and four native chickens had a single infection by *L. macleani*, whereas one fighting cock and three native chickens had double infection of *L. macleani* and microfilaria. Of note, infections by *L*. *macleani* were confirmed by combining microscopic and molecular analysis. Twelve native chickens had a single infection of *Leucocytozoon* sp. Seven fighting cocks and 20 native chickens had co-infection of *Leucocytozoon* sp. with other blood parasites, including one with *Leucocytozoon-Plasmodium*, six with *Leucocytozoon-Trypanosoma*, and 20 with *Leucocytozoon-*microfilaria. Furthermore, three native chickens had triple co-infection, one with *Leucocytozoon-Plasmodium*-*Trypanosoma* and two with *Leucocytozoon-Trypanosoma*-microfilaria.

The logistic regression (Table 2) indicated that male chickens had a 2.07 times higher risk of *Leucocytozoon* infection than female chickens (*p* = 0.391). The chickens raised in Roi-Et and Sakon Nakhon had a lower risk of *Leucocytozoon* infection than those raised in Kalasin (OR = 4.98×10⁻⁹ and 2.13×10⁻⁸, respectively; both *p* = 0.993).

**Table 2:** Logistic regression analysis of associations between prevalence of *Leucocytozoon* spp. and chicken sexes and localities.

	Odds ratio (OR)	95% CI	*p*-value*
Sex			
Female	Reference		
Male	2.07	−0.97 – 2.45	0.391
Localities			
Kalasin	Reference		
Roi-Et	4.98×10⁻⁹	NA – 150.09	0.993
Sakon Nakhon	2.13×10⁻⁸	NA – 172.12	0.993

*Fisher’s exact test.

### Morphological characteristics

**Figure 2:**
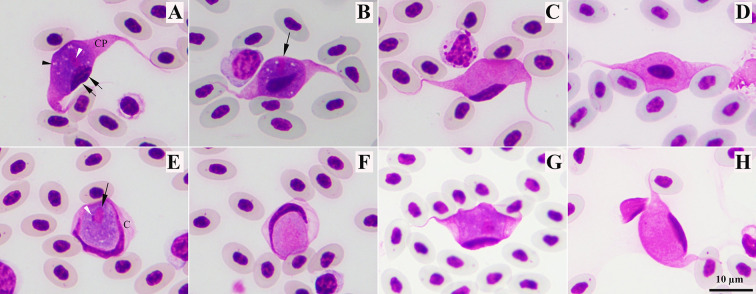
Gametocytes of *Leucocytozoon macleani*, lineages GALLUS17 (A–F) and lineages GALLUS63 (G–H). *Leucocytozoon macleani* gametocytes develop in two types of host cells, including fusiform (A–D, G–H) and roundish (E–F). Cytoplasm of macrogametocytes (A–B, E, G) usually contains small vacuoles (back arrowhead). Nucleus of macrogametocyte (arrow) contains nucleolus (white arrowhead). Two cytoplasmic processes (CP) present in fusiform host cells. Cytoplasm (C) of roundish host cells is largely occupied by macrogametocytes. Both fusiform and roundish host cells with microgametocytes have features similar to host cells with macrogametocytes. Nuclei of microgametocytes are diffuse and cytoplasm stains paler than in macrogametocytes. In fusiform host cell, macrogametocytes and microgametocytes usually displace the nucleus of host cell (double arrow) toward the periphery (A, C, G–H). In fusiform host cells, macrogametocytes and microgametocytes push nuclei aside, deforming them; the nuclei assume band-like form and lie peripherally like a band extending more than half of the circumference of gametocytes. Methanol-fixed, Giemsa-stained blood films. Scale bar = 10 μm.

Based on the morphological and molecular characteristics, only nine samples were identified as *L. macleani* single strain infection (Table 3). Of these nine *L. macleani* samples, only those with parasitaemia above 0.01% were included for further morphometric measurements. Gametocytes (both macrogametocytes and microgametocytes) developed in roundish (Figs. 2E, 2F) and fusiform (Figs. 2A–2D, 2G, 2H) host cells. Macrogametocytes contained a few small vacuoles (Fig. 2A). In fusiform cells, the nuclei of the host cells were usually pushed aside, and lay peripheral as cap-like structures (Fig. 2A, 2C, 2G, 2H). The length of the nuclei of the host cells in GALLUS17, and GALLUS63 was less than 1/3 of the circumferences of gametocytes (Table 4). Nucleus of the parasite (Fig. 2B) was variable both in form and position, with a prominent nucleolus (Fig. 2A). The cytoplasm was stained deep blue, which formed two processes at the ends of gametocytes (Fig. 2A). Length of the cytoplasmic processes was bigger than width.

**Table 3:** Species identification of *Leucocytozoon* found in Thai chickens (*Gallus gallus domesticus*).

Chickens (code)	Breed	Sex	Locality	Parasites	Parasite intensity (%)	Lineages	GenBank accession Nos.
KLS03	Fighting cock	Male	Kalasin	*Leucocytozoon macleani*	0.01	GALLUS17	PQ560893
KLS05	Fighting cock	Male	Kalasin	*Leucocytozoon macleani*	0.05	GALLUS17	PQ560895
KLS15	Native	Female	Kalasin	*Leucocytozoon macleani*	<0.01	GALLUS34	PQ560899
ROE03	Native	Male	Roi-Et	*Leucocytozoon* sp.*	0.00	ASIFLA01	PQ880117
ROE12	Native	Male	Roi-Et	*Leucocytozoon macleani*	<0.01	GALLUS17	PQ560906
ROE16	Native	Male	Roi-Et	*Leucocytozoon macleani*	0.03	**GALLUS63**	PQ560907
ROE17	Native	Male	Roi-Et	*Leucocytozoon macleani*	<0.01	GALLUS17	PQ560908
SKN07	Native	Female	Sakon Nakhon	*Leucocytozoon macleani*	0.08	GALLUS17	PQ560910
SKN08	Native	Female	Sakon Nakhon	*Leucocytozoon macleani*	0.16	GALLUS17	PQ560911
SKN14	Native	Male	Sakon Nakhon	*Leucocytozoon macleani*	<0.01	GALLUS17	PQ560916

*No gametocytes found in blood smear. Bold font is New lineage.

**Table 4:** Morphometry of gametocytes and host cells in two lineages of *Leucocytozoon macleani* from chickens in Thailand.

Features	Measurements (Min – Max (Mean ± SD))					
	Lineage GALLUS17				Lineage GALLUS63	
	Fusiform macrogametocytes (*n* = 35)	Fusiform microgametocytes (*n* = 35)	Roundish macrogametocytes (*n* = 20)	Roundish microgametocytes (*n* = 20)	Fusiform macrogametocytes (*n* = 10)	Fusiform microgametocytes (*n* = 10)
Parasite						
Length (μm)	**10.23–15.22 (13.19 ± 1.28)**	10.69–16.04 (13.29 ± 1.15)	8.79–13.57 (11.56 ± 1.35)	9.64–13.55 (12.01 ± 0.83)	**10.02–14.03 (11.93 ± 1.21)**	10.19–15.84 (12.46 ± 1.68)
Width (μm)	6.73–10.50 (8.57 ± 0.95)	6.25–10.07 (7.93 ± 0.91)	7.62–13.63 (10.11 ± 1.73)	7.90–10.63 (9.33 ± 0.73)	6.04–10.88 (8.65 ± 1.33)	7.11–10.32 (8.33 ± 1.06)
Perimeter (μm)	37.71–49.62 (43.97 ± 2.67)	39.86–51.68 (45.09 ± 3.01)	35.68–40.87 (37.71 ± 1.58)	33.81–39.93 (36.93 ± 1.39)	40.68–46.37 (43.34 ± 1.70)	41.30–52.95 (45.98 ± 3.56)
Area (μm^2^)	86.25–122.89 (106.65 ± 9.38)	79.94–116.38 (100.52 ± 9.35)	81.83–105.66 (93.27 ± 7.00)	78.92–99.59 (89.50 ± 6.31)	76.95–112.79 (101.24 ± 10.29)	91.54–114.02 (103.26 ± 7.33)
Parasite nucleus						
Length (μm)	3.38–8.53 (5.98 ± 1.39)	–	3.66–7.79 (5.00 ± 0.96)	–	4.27–6.69 (5.54 ± 0.87)	–
Width (μm)	1.10–3.14 (1.89 ± 0.48)	–	1.64–3.35 (2.60 ± 0.41)	–	1.26–2.77 (2.08 ± 0.46)	–
Area (μm^2^)	3.31–14.51 (8.90 ± 2.47)	–	6.64–17.23 (10.67 ± 2.27)	–	5.55–12.72 (9.39 ± 2.07)	–
Host–parasite complex						
Length (μm)	**37.30–64.18 (51.65 ± 6.98)**	**32.00–67.71 (50.66 ± 8.09)**	–	–	**31.94–51.64 (38.70 ± 6.69)**	**24.38–47.67 (34.84 ± 8.06)**
Width (μm)	10.31–13.63 (11.59 ± 0.74)	8.32–12.75 (10.93 ± 1.00)	–	–	8.67–12.89 (11.58 ± 1.32)	9.80–14.39 (11.56 ± 1.37)
Area (μm^2^)	**172.40–234.32 (198.60 ± 15.63)**	136.94–232.33 (187.19 ± 23.56)	147.50–215.53 (181.84 ± 17.81)	145.98–199.29 (172.25 ± 11.15)	**137.05–209.79 (183.71 ± 23.93)**	150.89–204.11 (181.07 ± 17.20)
Diameter (μm)	–		12.92–16.97 (14.79 ± 1.05)	13.15–15.77 (14.66 ± 0.86)	–	–
Host nucleus						
Length (μm)	6.89–12.38 (9.76 ± 1.22)	6.93–11.20 (8.96 ± 1.07)	20.03–32.14 (24.57 ± 3.45)	20.32–34.38 (27.65 ± 4.03)	7.90–11.35 (9.52 ± 0.98)	6.38–11.61 (8.79 ± 1.38)
Width (μm)	1.78–4.49 (2.90 ± 0.51)	2.08–4.04 (3.13 ± 0.45)	1.85–3.77 (2.45 ± 0.50)	1.90–3.24 (2.34 ± 0.36)	2.20–3.22 (2.86 ± 0.33)	2.72–3.60 (3.19 ± 0.27)
Area (μm^2^)	18.13–27.67 (23.31 ± 2.80)	13.77–30.96 (23.14 ± 3.92)	27.41–52.58 (39.39 ± 6.56)	34.32–51.34 (42.17 ± 3.96)	19.08–32.22 (23.72 ± 3.80)	21.29–26.99 (24.42 ± 1.98)
Cytoplasmic processes						
Length (μm)	**11.59–26.15 (20.29 ± 3.88)**	**9.99–25.47 (19.34 ± 4.17)**	–	–	**10.04–20.84 (13.70 ± 3.40)**	**5.95–19.33 (11.19 ± 4.32)**
Width (μm)	4.32–10.32 (6.91 ± 1.47)	**4.07–8.23 (6.26 ± 1.13)**	–	–	5.02–10.34 (7.74 ± 1.55)	**6.74–10.17 (8.56 ± 1.10)**
Area (μm^2^)	**20.03–50.14 (35.00 ± 7.73)**	15.87–55.84 (33.33 ± 10.86)	–	–	**18.89–38.96 (28.13 ± 7.27)**	14.63–47.54 (28.44 ± 9.31)

Bold is significant difference.

In roundish cells, nuclei of the host cells were deformed, pushed peripherally and lied usually as a band. Host cell nuclei covered more than half of the circumference of gametocytes. Nucleus had a prominent nucleolus (Fig. 2E). The cytoplasm of roundish host cell was largely replaced by gametocytes, which usually was found around gametocyte as pale margin of variable form (Fig. 2E).

Microgametocytes of *L. macleani* showed diffused nucleus that was variable in location. The cytoplasm was stained pale purple (Figs. 2C, 2D, 2F, 2H). In fusiform cells, two cytoplasmic processes were seen, and the cytoplasm rarely contained vacuoles. Length of the cytoplasmic processes was bigger than width. Host cell nucleus was usually pushed aside; it lied periphery (Fig. 2C). Nucleus of fusiform host cell had the length less than 1/3 of the circumferences of gametocytes (Table 4).

Roundish microgametocytes were rarely found. Gametocyte displaced the nucleus of host cell toward the periphery (Fig. 2F). Nucleus of host cell had the band-shaped appearance with the length greater than half of the circumference of gametocyte. The cytoplasm of roundish host cell was largely replaced by gametocytes, which was similar to that of macrogametocytes.

Morphometric analysis showed that some parameters of fusiform and roundish macrogametocytes of *L. macleani* GALLUS17 (Table 4) fell within the min – max range of *L. macleani* from the previous reports (Table 5). However, the size of the nucleus of roundish host cells in *L. macleani* GALLUS17 (mean ± SD = 24.57 ± 3.45 μm; and min – max = 20.03 – 32.14 μm) was greater than reported in previous studies (8.20 – 22.20 μm). In regard to *L. macleani* GALLUS63, morphometrics of fusiform gametocytes fell within the min – max range of *L. macleani* from the previous reports.

**Table 5:** Morphometry of gametocytes and host cells of *Leucocytozoon macleani* from previous reports.

Features	Measurements					
	Valkiūnas [58]				Sacchi and Prigioni [45]	
	Fusiform macrogametocytes (*n* = 40)		Roundish macrogametocytes (*n* = 31)		Fusiform macrogametocytes (*n* = 10)	Fusiform microgametocytes (*n* = 10)
	Mean ± SD	Min – Max	Mean ± SD	Min – Max	Mean ± SD	Mean ± SD
Parasite						
Length (μm)	17.70 ± 1.40	12.20–24.40	13.10 ± 1.10	10.20–15.80	17.90 ± 1.40	13.70 ± 2.00
Width (μm)	11.00 ± 1.30	6.00–15.40	12.00 ± 0.90	10.00–15.00	9.60 ± 1.40	7.90 ± 1.50
Perimeter (μm)	–	–	–	–	–	–
Area (μm^2^)	–	–	–	–	113.90 ± 17.60	84.20 ± 14.70
Parasite nucleus						
Length (μm)	4.0 ± 0.4	2.20–7.00	4.00 ± 0.80	2.60–5.40	–	–
Width (μm)	3.70 ± 0.40	1.40–5.30	3.00 ± 0.80	1.00–4.40	–	–
Area (μm^2^)	–	–	–	–		
Host nucleus						
Length (μm)	13.90 ± 1.00	8.80–16.80	15.80 ± 3.00	8.20–22.20	11.80 ± 1.80	10.80 ± 2.00
Width (μm)	–	–	–	–	2.70 ± 0.60	3.00 ± 0.70
Area (μm^2^)	–	–	–	–	22.30 ± 6.90	21.50 ± 3.70

Additionally, length of cytoplasmic processes of macrogametocytes and microgametocytes in *L. macleani* GALLUS63 were significantly shorter than those of *L. macleani* GALLUS17 (Table 4). The width of cytoplasmic processes in microgametocytes of GALLUS63 was significantly broader than that of GALLUS17, *p* < 0.05. The area of cytoplasmic processes in macrogametocytes of GALLUS63 was significantly smaller than that of GALLUS17, *p* < 0.05. The length of the host-parasite complex of macrogametocytes and microgametocytes in GALLUS63 was significantly smaller than that of GALLUS17, *p* < 0.05. The area of the host-parasites complex of macrogametocytes in GALLUS63 was significantly smaller than that of GALLUS17, *p* < 0.05.

### Molecular characteristics and phylogenetics

The sequence analysis from 54 samples revealed that most chickens were co-infected with multiple *Leucocytozoon* lineages (*n* = 44). Only ten chickens were infected by a single *Leucocytozoon* lineage, which were identified as four lineages as GALLUS17, GALLLUS34, GALLUS63, and ASIFLA01 (Table 3). The lineage GALLUS63 was the novel lineage and was isolated from a chicken (samples: ROE16). Additionally, the most common lineage found in this study was the GALLUS17, which was found in seven chickens (both fighting cock and native chickens) raised in Kalasin (samples: KLS03 and KLS05), Roi-Et (samples: ROE12 and ROE17) and Sakhon Nakhon (samples: SKN07, SKN08 and SKN14).

**Figure 3:**
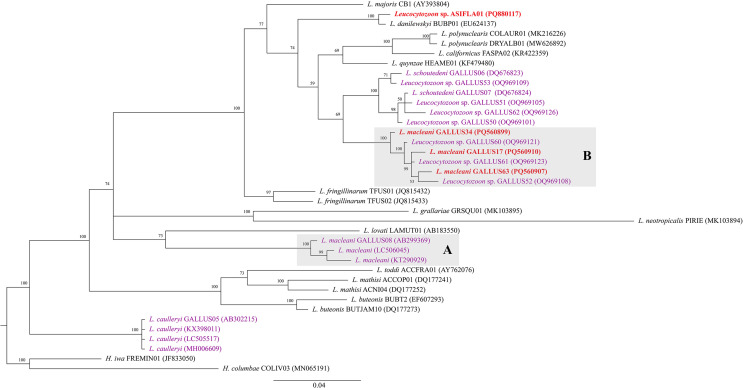
Bayesian phylogeny constructed by using the cytochrome *b* sequences (479 bp) of *Leucocytozoon* parasites, including four sequences isolated from village chickens in Thailand (orange bold text) and other sequences deposited in the MalAvi database. Other *Leucocytozoon* lineages isolated from chickens are given in purple. Clade A contains sequences of *Leucocytozoon macleani* reported in other studies, while clade B contains sequences of *L. macleani* isolated from this study. MalAvi lineage codes and GenBank accession number are given after species name. Node values (in percentage) indicate the posterior clade probabilities.

The Bayesian phylogenetic inference (Fig. 3) of *cytb* gene revealed that *L. macleani* lineages GALLUS17, GALLUS34, and GALLUS63 grouped separately from *L. macleani* GALLUS08 isolated from chicken in Malaysia and other two *L. macleani* isolated from chickens in Thailand and Malaysia. However, our three *L. macleani* sequences GALLUS17, GALLUS34, and GALLUS63 grouped with other *Leucocytozoon* spp. lineages obtained from chickens in Thailand, GALLUS52, GALLUS60, and GALLUS61. The genetic homology in this clade was 97.30%–99.57%. Furthermore, our *L. macleani* (clade B) had 79.85%–82.07% genetic similarity with other *L. macleani* (clade A), 85.20%–86.84% genetic similarity with *Leucocytozoon caulleryi* and 90.35%–92.77% genetic similarity with *Leucocytozoon schoutedeni*. Additionally, one *Leucocytozoon* sequence isolated in this study was identified as lineage ASIFLA01. This lineage was grouped together with *Leucocytozoon danilewskyi* lineage BUBP01, which was previously reported from the Eurasian eagle-owl (*Bubo bubo*) in Spain (Fig. 3). Our *Leucocytozoon* sp. ASIFLA01 showed 98.70% similarity to *L. danilewskyi* BUBP01.

## Discussion

Blood parasites in poultry are often neglected; however, their prevalence can be especially high in hot and humid regions, such as Thailand. According to the reported exo-erythrocytic stages of *L. macleani*, merogony takes places in hepatocytes, brain capillary endothelial cells, and renal tubular cells [58, 62]. Generally, the pathogenicity of most *Leucocytozoon* species appears to be associated with the presence or absence of megalomeronts, which are often encapsulated by fibrotic tissue and surrounded by mixed inflammatory infiltrates, including erythrocytes, plasma cells, macrophages, and heterophils. Finally, these megalomeronts might undergo necrosis and calcification [2].

Atkinson *et al*. (1991) also explained that *Leucocytozoon* species can cause severe anaemia through the destruction of infected blood cells by the reticuloendothelial cells in the spleen, as well as by the destruction of uninfected erythrocytes by anti-erythrocyte factors appearing in the serum during acute infection. However, the megalomeronts were not found in *Leucocytozoon macleani* [62]. Additionally, due to high prevalence of *Leucocytozoon* parasites and the frequent occurrence of multiple-strain infections in this study, further targeted investigation is required before any conclusions on pathology of *L. macleani* infections in Thai chickens. As our first step in research of chicken leucocytozoonosis, this study only explained detailed molecular and morphological characteristics of *L. macleani* that are prevalent in this area. The finding showed that prevalence of leucocytozoonosis detected by PCR (90%) was slightly higher than that of microscopy (85%). The authors suggested using microscopic method in routine laboratory diagnosis of chicken leucocytozoonosis, as it is an inexpensive method suitable for use in underdeveloped and developing countries.

The prevalence of leucocytozoonosis was high in Thai domestic chickens (*Gallus gallus domesticus*) similarly to the previous reports in the country [5, 25]. That highlighted the need for continuous monitoring and challenges for the elimination of leucocytozoonosis. Additionally, it underscored the importance of further investigation to identify potential transmission of leucocytozoonosis agents to the larger commercial poultry production sector. Furthermore, it might be worth conducting further studies to precisely measure the economic impact of leucocytozoonosis as well, particularly due to damage to internal organs by tissue stages, which remain insufficiently investigated both in *L. schoutedeni* and *L. macleani*, especially in juvenile birds.

The majority of infected chickens (42 out of 51 infected chickens) had moderate level of parasitaemia (>0.01% to 1%). The level of parasitaemia might be associated with host population density, as a higher density could increase the transmission rate and promote infection by different strains, ultimately leading to increased parasitaemia [33]. Thus, implementing flock size manipulation might reveal evidence that supports this assumption. On the other hand, the intensity of parasitaemia might depend on circadian rhythms. Chagas *et al*. [8] described the circadian patterns in avian blood parasites, including *Plasmodium*, *Leucocytozoon* species, *Trypanosoma* species and microfilariae, with *Leucocytozoon* species peaking mainly during the evening and night. Additionally, the level of parasitaemia might be influenced by exposure to potential vectors. In a previous study in Boreal Owls (*Aegolius funereus*) infected by *Leucocytozoon* parasites, it was shown that males, which hunt prey for their mate and nestlings during incubation, had higher levels of parasitaemia than females, which typical remain in cavity nests [49]. Although chickens with moderate parasitaemia were likely invulnerable to *Leucocytozoon* infection, these infections cannot be neglected, mainly because in favourable conditions, these infections might lead to severe illness. We assumed that parasitaemia intensity might be related to the tissue merogony stage and the patterns of persistence in avian hosts. In another haemosporidian parasite, *Haemoproteus*, the parasitaemia level did not necessarily correlate with the abundance of tissue merogony, and the presence of parasitaemia was not an indicator of the existence of tissue merogony [12]. However, this phenomenon remains insufficiently studied in chickens, highlighting the need for further research.

Multiple blood parasites and multiple *Leucocytozoon* lineage infections were frequently observed in this study, with microscopic examination revealing the presence of up to three different blood parasites in a single host. Blood parasites are transmitted by various blood-sucking insects. For example, *L. macleani* and *L. schoutedeni* are transmitted by blackflies, while *L. caulleryi* is transmitted by biting midges [58]. *Plasmodium gallinaceum* and *P. juxtanucleare* are known to be transmitted by *Culex* mosquitoes [5, 42], whereas *Trypanosoma* spp. are carried by mosquitoes, hippoboscid flies, black flies, biting midges, and mites [34, 38, 51, 55, 69]. Microfilariae were transmitted by blackflies, biting midges, mosquitoes, and tabanids [1, 16, 40, 47]. Isolation of multiple parasites from a single bird in this study indicated the presence of diverse vectors and transmission of different vector-borne pathogens in the area; however, this was not investigated in the current study.

Based on analysis of electropherogram, the presence of double peaks in *cytb* sequences indicated infections by multiple *Leucocytozoon* lineages. This approach for identification of mixed-lineages infection has been used in several studies, including a study of *Leucocytozoon* sp. and *Plasmodium* sp. in *Gallus gallus domesticus* [5]; a study of *Leucocytozoon* sp. in Passeriformes birds [15]; a study of *Leucocytozoon* sp., *Plasmodium* sp., and *Haemoproteus* sp. in Accipitriformes birds [19]; a study of haemosporidian parasites in Accipitriformes and Strigiformes birds [37, 39]; a study of haemosporidian parasites in Passeriformes birds [6]; and a study of haemosporidian parasites in Passeriformes, Coraciiformes, Columbiformes, and Piciformes birds [24]. The presence of these mixed infections make lineage description based on the MalAvi nomenclature [4] impossible. In this study, only single lineage infections were assigned the lineage.

Sequencing of *cytb* gene fragments revealed that most chickens were infected with multiple *Leucocytozoon* lineages, except for ten chickens. This finding highlighted the importance of careful identification and description of *Leucocytozoon* in chickens. For this reason, it was suggested to combine microscopy and molecular techniques for identification of *Leucocytozoon* parasites for species description. Although various compounds (e.g., clopidol, primaquine, halofuginone polystyrene sulfonate, and sulfamonomethoxine and ormetoprim combinations) have been used to treat *Leucocytozoon* infections, their efficacy remains debated [68]. Furthermore, Zhao *et al*. (2016) found that sulfaquinoxaline or pyrimethamine had no or limited effects on *L. macleani* infections. Sulfamonomethoxine at concentration of 30 and 40 ppm can be used for the treatment of *L. caulleryi* leucocytozoonosis in poultry [29]. Sulfamonomethoxine is a sulfonamide antibiotic inhibiting dihydropteoate synthetase (DHPS), which is an important enzyme for the folate pathway of bacteria and primitive eukaryotes [67]. The products from this pathway are essential for DNA synthesis [22], which might influence asexual multiplication at the merogony stage of leucocytozoids.

Nine out of ten chickens with single infection were infected by *Leucocytozoon* parasites that developed both in fusiform and roundish host cells, which were the characteristics of *L. macleani*. In contrast, *L. caulleryi* and *L. schoutedeni* developed exclusively in roundish host cells [58]. *Leucocytozoon macleani* fusiform macrogametocytes exhibited characteristic features resembling those previously described, such as cytoplasm containing small vacuoles, a host cell nucleus extending less than 1/3 of the circumference of gametocyte, and the cytoplasmic processes longer than their width [45, 58]. However, in roundish cells, the nucleus extended beyond half of the gametocyte’s circumference. This differed from the previous description, in which nucleus of roundish host cell extended less than half of the gametocyte’s circumference [58]. From the morphometric point of view, it was found that the length of nucleus of roundish host cells in this study (Table 3) was longer than the previous report (Table 4). Additionally, there was a significant difference in the length of cytoplasmic processes and length of host-parasite complex in *L. macleani* lineages. These distinct characteristics might provide evidence for a species complex within *L. macleani*, a phenomenon previously described in *Leucocytozoon toddi*, which developed within fusiform host cells of diurnal raptors [61].

More recently, Valkiūnas *et al*. (2010) identified two populations of *L. toddi* with different lengths of cytoplasmic processes, supported by the genetic divergence of the *cytb* gene between these two groups. Together with the different avian host, *L. toddi* in these two populations was split into *Leucocytozoon mathisi* and *Leucocytozoon buteonis*. In this study, the difference in morphologic characteristics (Fig. 2) was supported by phylogenetic analysis (Fig. 3) and genetic divergence among *cytb* sequences of our isolated *L. macleani* lineages, 1.3% to 2.19% genetic distances. Thus, *L. macleani* GALLUS17 and GALLUS63 (new lineage identified in this study) might represent a case of cryptic speciation (these two lineages showed 1.74% genetic distance). However, it was not within the scope of this study to describe a new subspecies, which requires further investigation, including type of host cell infected by the parasite, parasite biology, and detailed molecular information [15].

This study revealed the common *L. macleani* lineages in Thailand, which was lineage GALLUS17. Previously, this lineage was also found in Southern Thailand [5]. Thus, *L. macleani* GALLUS17 might be the important leucocytozoonosis agent causing the negative impact in chickens in a wider geographical area. Together with our finding of the existence of a new lineage, *L. macleani* GALLUS63 and previous reports of several lineages of both described and non-described species in Thailand [52], it can be assumed that there is high genetic diversity of *Leucocytozoon* sp. in the country. This study assumed uncertainty regarding the pathology of *L. macleani* infection in Thai chicken due to the frequent occurrence of multiple *Leucocytozoon*-strain infections. On a different note, *L. macleani* had been reported to be of low virulence [48, 58], which might enable the parasite to produce multiple successive generations and gradually drive genetic change over the time. The relationship between lineage diversity and evolution in haemosporidian parasites was previously discussed by Huang *et al*. [21]. Thus, the authors suggested that *L. macleani* is a suitable model organism for such research, due to its high lineage diversity and the ease of conducting experimental studies with its vertebrate host.

Additionally, this study was able to retrieve one sequence of owl *Leucocytozoon* sp. from chicken blood, which was the *Leucocytozoon* sp. lineage ASIFLA01 (GenBank accession No: PQ880117). This lineage ASIFLA01 was originally described from *Leucocytozoon* sp. isolated from Short-eared Owl (*Asio flammeus*) in Japan (GenBank accession No: LC230137) [23]. A previous study also reported the detection of wild bird haemosporidian DNA in chicken blood, and our study supported that finding. Chatan *et al*. [10] reported the *Plasmodium* sp. lineage ACCBAD01, FANTAIL01, and ORW1, which were originally described from Shikra (*Accipiter badius*, GenBank No: JN639001), Australian Rufous Fantail (*Rhipidura rufifrons*, GenBank No: AY714196), and Oriental Reed Warbler (*Acrocephalus orientalis*, GenBank No: AF254963), respectively. This phenomenon was considered an abortive infection, where the parasite invaded the wrong host, but failed to complete its entire life cycle [62].

Last but not least, Bayesian phylogenetics revealed two clades of *L. macleani* (Fig. 3). Clade A, containing *L. macleani* isolated from chickens raised in Malaysia and Thailand, showed 17.93% to 20.15% genetic distance from clade B containing our *L. macleani*. Furthermore, clade A was grouped closely with *Leucocytozoon lovati* lineage LAMUT01 reported by Sato *et al*. [46], with 18.48% to 19.03% genetic distances, whereas clade B had 16.84% to 17.93% genetic distance from *L. lovati*. Morphologically, *L. macleani* and *L. lovati* were very similar [58]. The length of the host cell-parasite complex in *L. lovati* ranged from 22.0 to 60.8 μm (mean ± SD: 42.7 ± 8.94 μm) for macrogametocytes and 5.6 to 60.8 μm (mean ± SD: 45.9 ± 9.17 μm) for microgametocytes [17]. In this case, the authors found high genetic distance between *L. macleani* and *L. lovati*. However, based on Bayesian phylogenetic analysis, *L. lovati* might be able to infect chickens and could be a novel pathogen of poultry.

## Conclusion

This study revealed a high prevalence of *Leucocytozoon* infections in chickens in Thailand, calling for more research on these pathogens in poultry. It is the first report describing lineages in *Leucocytozoon macleani* (synonym *Leucocytozoon sabrazesi*) in Southeast Asia, following the MalAvi database nomenclature, together with the detailed morphological characteristics of gametocytes and their host cells. A new lineage of *L. macleani* was identified (GALLUS63). Furthermore, our findings suggested potential cryptic speciation within *Leucocytozoon macleani*. This study identified many multiple *Leucocytozoon*-strain infections, highlighting the importance of careful identification and description of these parasites in chickens. Altogether, this information can serve as a valuable reference for routine veterinary laboratory diagnostics and future investigation. Research on the exo-erythrocytic development of chicken leucocytozoids remains in its infancy but is essential for better understanding pathology during poultry leucocytozoonosis.
